# Host immune responses after hypoxic reactivation of IFN-γ induced persistent *Chlamydia trachomatis* infection

**DOI:** 10.3389/fcimb.2014.00043

**Published:** 2014-04-16

**Authors:** Stefan Jerchel, Inga Kaufhold, Larissa Schuchardt, Kensuke Shima, Jan Rupp

**Affiliations:** ^1^Institute of Medical Microbiology and Hygiene, University of LübeckLübeck, Germany; ^2^Medical Clinic III/Infectious Diseases, University Hospital of Schleswig-HolsteinLübeck, Germany

**Keywords:** *Chlamydia trachomatis*, persistence, hypoxia, reactivation, immune response

## Abstract

Genital tract infections with *Chlamydia trachomatis* (*C. trachomatis*) are the most frequent sexually transmitted disease worldwide. Severe clinical sequelae such as pelvic inflammatory disease (PID), tubal occlusion, and tubal infertility are linked to inflammatory processes of chronically infected tissues. The oxygen concentrations in the female urogenital tract are physiologically low and further diminished (0.5–5% O_2_, hypoxia) during an ongoing inflammation. However, little is known about the effect of a low oxygen environment on genital *C. trachomatis* infections. In this study, we investigated the host immune responses during reactivation of IFN-γ induced persistent *C. trachomatis* infection under hypoxia. For this purpose, the activation of the MAP-kinases p44/42 and p38 as well as the induction of the pro-inflammatory cytokines IL-1β, IL-6, IL-8, and MCP-1 were analyzed. Upon hypoxic reactivation of IFN-γ induced persistent *C. trachomatis* infection, the phosphorylation of the p44/42 but not of the p38 MAP-kinase was significantly diminished compared to IFN-γ induced chlamydial persistence under normoxic condition. In addition, significantly reduced IL-6 and IL-8 mRNA expression levels were observed for reactivated *Chlamydiae* under hypoxia compared to a persistent chlamydial infection under normoxia. Our findings indicate that hypoxia not only reactivates IFN-γ induced persistent *C. trachomatis* infections resulting in increased bacterial growth and progeny but also dampens inflammatory host immune signaling responses that are normally observed in a normoxic environment.

## Introduction

*Chlamydia trachomatis* (*C. trachomatis*) is an obligate intracellular pathogen and the most frequent sexually transmitted bacterium worldwide. In the United States ~1.5 million infections were reported in 2011 (Centers for Disease Control and Prevention, 2012). While most of the infections occur without symptoms, a symptomatic manifestation of urogenital chlamydial infection can be observed in ~30% of the patients. In a subset of female patients, ascending genital tract infections cause disease such as salpingitis, pelvic inflammatory disease (PID), or tubal infertility (Peipert, 2003; Mardh, 2004). Chlamydial pathology is attributed to severe inflammatory processes leading to scarring and loss of functional epithelial tissue (Peipert, 2003; Mardh, 2004). During the infection, different pro-inflammatory cytokines such as Interleukin (IL)-1, IL-6, and IL-8 are induced and thought to affect the disease outcome (Rasmussen et al., 1997; Hanada et al., 2003; Buchholz and Stephens, 2007; Hvid et al., 2007). Reoccurrence or a chronic infection with *C. trachomatis* are discussed to be central mediators of disease progression and final outcome (Dean et al., 2000). However, it is not clear whether chlamydial reoccurrence after a symptomatic episode with/without antibiotic treatment occurs mainly due to reinfection transferred from the sexual partner or due to reactivation of persistent *C. trachomatis* from a silent state (Golden et al., 2005; Geisler, 2007). Persistence describes a non-infectious but viable developmental stage. In the *in vitro* chlamydial persistence models, the infection is characterized by an altered intracellular inclusion morphology that is accompanied by reduced chlamydial progeny and increased cell survival compared to actively replicating pathogens (Hogan et al., 2004). Chlamydial persistence can be induced through various stimuli including interferon-γ (IFN-γ), treatment with sub-inhibitory concentrations of antibiotics or iron depletion (Wyrick, 2010). Although the induction of persistent *Chlamydiae* has been extensively studied *in vitro*, data showing persistently infected urogenital tissues in diseased females are still missing, hence characteristics of persistent *in vivo* urogenital *C. trachomatis* infections in humans were not observed yet. Regarding this, persistence defining properties are only based on *in vitro* experiments and might be different *in vivo*. Persistent chlamydial infection of the urogenital tract *in vivo* have so far only been shown in a *C. muridarum* mice infection model (Phillips et al., 2012). Phillips et al. could show a reduced number of infectious *Chlamydiae* in persistent infection while pre-16s rRNA expression was not changed, indicating a viable but not infectious chlamydial form (Phillips et al., 2012). Further, they could show chlamydial inclusions with abnormal reticulate bodies but without elementary bodies (EBs) via transmission electron microscopy (Phillips et al., 2012). These findings were in line with the observations of *in vitro* persistence models.

We and others could show that persistent *C. trachomatis* are less susceptible to currently available first-line antimicrobials which presumably could result in clinical treatment failures (Reveneau et al., 2005; Phillips et al., 2012; Shima et al., 2013) and might favor *C. trachomatis* survival within its biological niche thereby inducing chronic infections. In this study, we focused on the IFN-γ induced persistence of *C. trachomatis* as the most extensively studied model in the past (Beatty et al., 1993, 1994; Roth et al., 2010). Based on a previous observation that anti-chlamydial activity of IFN-γ is reduced in a low oxygen environment allowing persistent *Chlamydiae* to reactivate and proliferate (Roth et al., 2010), we wondered about the host immune responses that are induced during this process.

## Methods

### Epithelial cell culture and *C. trachomatis* infection

A total of 2.5 × 10^5^ HeLa-229 cells were cultured with 5 ml RPMI 1640 (PAA Laboratories, Cölbe, Germany) supplemented with 5% FBS (PAA Laboratories), 100 mg/L L-glutamine (PAA Laboratories), 1× non-essential amino acids (PAA Laboratories) with or without 5 U/ml IFN-γ (Peprotech, Hamburg, Germany) in a 6-well plate and incubated for 24 h under normoxia at 37°C, 20% O_2_, 5% CO_2_. IFN-γ was present over the whole experiment. After incubation, HeLa-229 cells were infected with 2 inclusion forming units (IFUs)/cell of *C. trachomatis* serovar D and centrifuged for 60 min at 700 × *g*. After 24 h incubation under normoxia, persistent *C. trachomatis* infected cells were further cultivated either in normoxic or hypoxic incubators (37°C, 2% O_2_, 5% CO_2_) (Toepffer Lab Systems, Goeppingen, Germany) for additional 2 and 3 days (d). The medium was exchanged every second day with medium containing 5 U/mL IFN-γ. Hypoxic samples were cultivated in preconditioned medium that was incubated under hypoxic conditions for 12 h before medium exchange.

### Chlamydial recovery

The burden of infectious *C. trachomatis* EBs after intracellular development under normoxic and hypoxic conditions was determined by titration experiments as described before (Beatty et al., 1993). In brief, infected cell monolayers were harvested and disrupted by glass beads. Disrupted cells including *C. trachomatis* were inoculated in serial dilutions on confluent HEp-2 cell monolayers in DMEM supplemented with 10% FBS, 100 mg/L L-glutamine, 1× non-essential amino acids under normoxia. Development of chlamydial inclusions was analyzed 48 h post infection (h p.i.) using an anti-*Chlamydia*-LPS antibody (kindly provided by H. Brade; Research Centre Borstel, Borstel, Germany) together with a secondary FITC-labeled anti-mouse antibody (Dako, Glostrup, Denmark). Recovered *C. trachomatis* were calculated by observation of 10 high-power fields (20× magnification). Infectious progeny under different conditions was determined by calculating the absolute IFUs. All data are the average from seven independent experiments and the error bars represent the standard error of the mean (s.e.m.).

### Fluorescence microscopy

To analyze *C. trachomatis* inclusion morphology after 2 and 3 d infection, *C. trachomatis* infected cells were grown on coverslips. After fixation with methanol, cells were stained with FITC-labeled monoclonal anti-*Chlamydia*-LPS antibody and evans blue (Oxoid, Cambridgeshire, UK) and morphology was analyzed by a fluorescence microscope (Keyence, Osaka, Japan). To determine the inclusion size 10 inclusions/condition from five different experiments (total 50 inclusions/condition) were measured by using the BZ-II-Analyzer (Keyence, Osaka, Japan).

### Western blot analysis

For determination of phospho-p38/-p44/42 (Cell Signaling, Danvers), cells were prepared with western blot lysis buffer (125 mM Tris-HCl pH 7.8, 20% glycerol, 4% SDS (sodium dodecyl sulfate), 0.1 M dithiothreitol, bromphenol blue, Sigma Aldrich, St. Louis) at the indicated time points. Samples were analyzed by SDS-Polyacrylamide gel electrophoresis (SDS-PAGE). Afterwards, proteins were transferred to nitrocellulose membranes (Whatman Inc., Florham Park, NJ). Membranes were blocked with TBS (0.1% Tween)/5% fat-free skimmed milk and incubated with the respective antibodies. For detection, a horseradish peroxidase-linked anti-mouse IgG antibody (Cell Signaling) and enhanced chemiluminescence substrate (Thermo Fisher Scientific, Rockford, IL) were used. Images were acquired by Fusion FX7 (Vilber Lourmat, Eberhardzell, Germany) and the density of each band was measured by Bio-1D software (Vilber Lourmat). Equal loading and blotting efficiency were verified by an anti-β-actin antibody and pre-stain marker (Cell Signaling). All data are the average from seven independent experiments and the error bars represent the standard error of the mean (s.e.m.).

### Analysis of mRNA expression

Total RNA was isolated using the NucleoSpin RNA II kit (Macherey-Nagel, Dueren, Germany) and transcribed into cDNA by the First-Strand PCR kit (Roche, Basel, Switzerland). PCR amplification was performed by using the LightCycler Detection System (Roche). Relative quantification of IL-1β (forward TCCCCAGCCCTTTTGTTGA, reverse TTAGAACCAAATGTGGCCGTG), IL-6 (forward CCTTCCAAAGATGGCTGAAA, reverse CAGGGGTGGTTATTGCATCT), IL-8 (forward CCAGGAAGAAACCACCGGA, reverse GAAATCAGGAAGGCTGCCAAG), MCP-1 (forward CATTGTGGCCAAGGAGATCTG, reverse CTTCGGAGTTTGGGTTTGCTT) mRNA expression was performed against 18S rRNA (forward TCAAGAACGAAAGTCGGAGG, reverse GGACATCTAAGGGCATCACA) and normalized to the respective mRNA expression levels in normoxic non-infected cells by using the 2^−ΔΔ*CT*^ method (Livak and Schmittgen, 2001). All data are the average from seven independent experiments and the error bars represent the standard error of the mean (s.e.m.).

### Statistical analysis

Data are indicated as mean ± s.e.m. Statistical analysis was performed with the tailed, unpaired Student *t*-test. *p*-values ≤ 0.05 were considered as statistically significant.

## Results

### Hypoxic reactivation of persistent *C. trachomatis*

**Figure 1:**
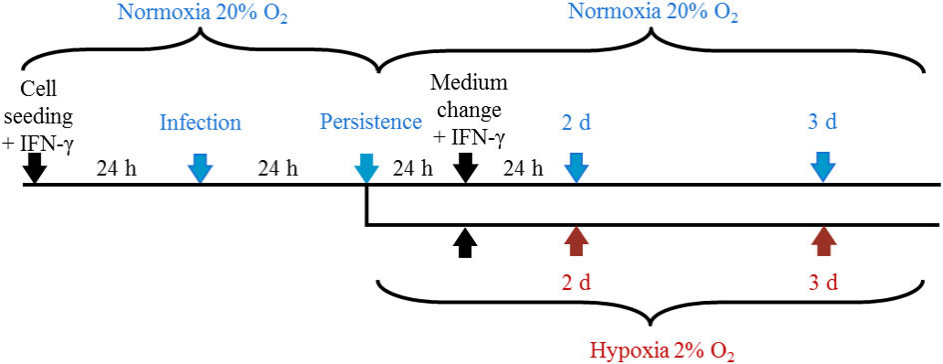
**Representation of the experimental setup.** Cells were cultured under normoxia for 24 h in the presence of IFN-γ (5 U/mL) and were infected with *C. trachomatis* (2 IFU/cell) the following day. After 24 h, cells were further incubated under normoxia (20% O_2_) or transferred to hypoxic (2% O_2_) conditions with constant IFN-γ supplementation before samples were collected at the indicated time points.

**Figure 2:**
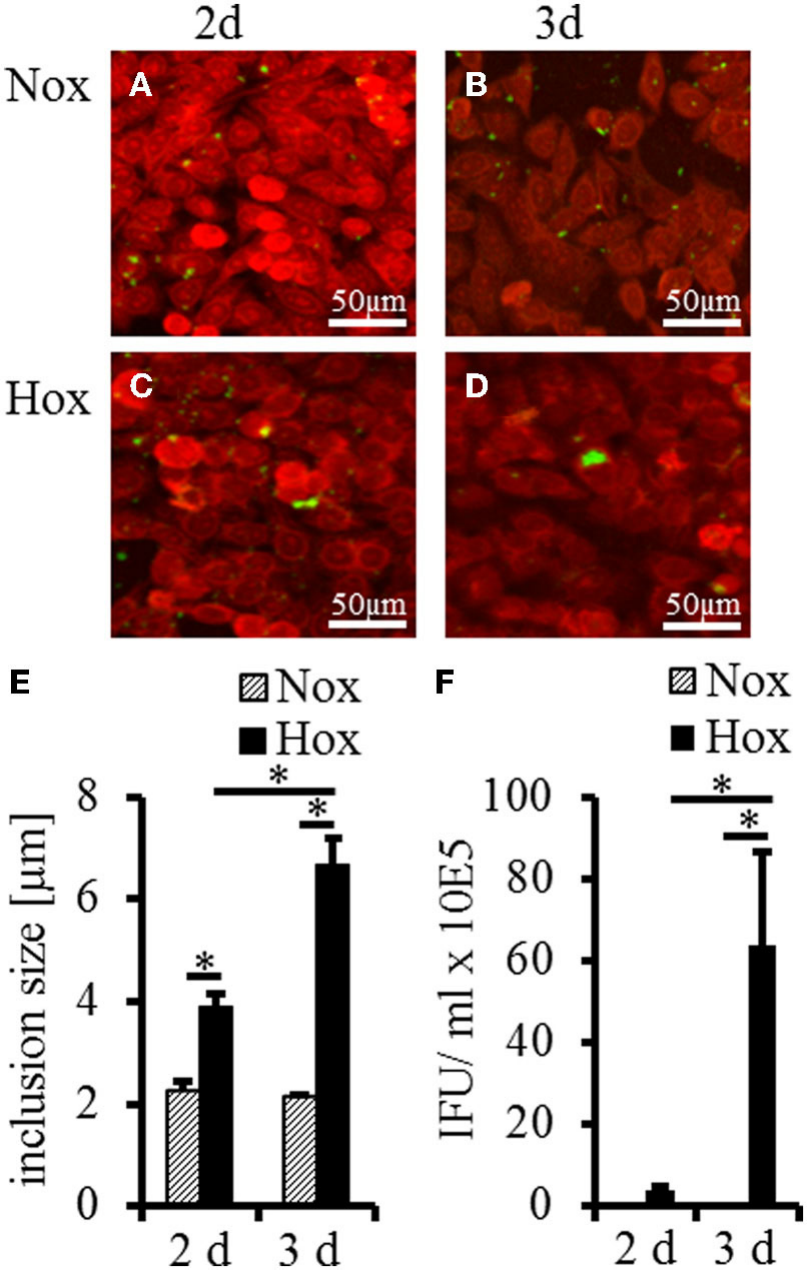
**Recovery analysis of IFN-γ treated *C. trachomatis* infected cells under normoxic and hypoxic conditions.** Immunofluorescence staining shows reactivation of *C. trachomatis* (green) in HeLa-229 cells (red) under hypoxic (Hox) conditions **(C,D)** but not under normoxic (Nox) conditions **(A,B)** after 2 and 3 d, respectively. Quantitative analysis of the inclusion size showed significant larger inclusion after 2 and 3 d under hypoxic condition compared with normoxic condition **(E)**. Quantitative analysis of the infectious progeny of IFN-γ treated *C. trachomatis* after incubation for up to 3 d under normoxic and hypoxic conditions **(F)** (*n* = 7, mean ± s.e.m., ^*^p ≤ 0.05).

To determine whether IFN-γ induced persistent *C. trachomatis* D could be reactivated in HeLa-229 cells under hypoxia, we used the experimental setup displayed in Figure 1. After transfer of persistent *C. trachomatis* in a hypoxic environment (2% O_2_), reactivation of chlamydial growth was observed by a significant increase in the inclusion size compared to cultivation under normoxic conditions (Figures 2A–E). In accordance, the amount of recoverable infectious *C. trachomatis* significantly increased after 3 days cultivation in hypoxia, whereas no increase was observed under normoxic conditions (Figure 2F). These observations confirm previous findings of reactivated *C. trachomatis* L2 in HEp-2 cells (Roth et al., 2010) and indicate that IFN-γ treatment under hypoxia is less effective to maintain *C. trachomatis* in a persistent state.

### Analysis of MAP-kinase p44/42 and p38 phosphorylation in reactivated *C. trachomatis* infection under hypoxia

**Figure 3:**
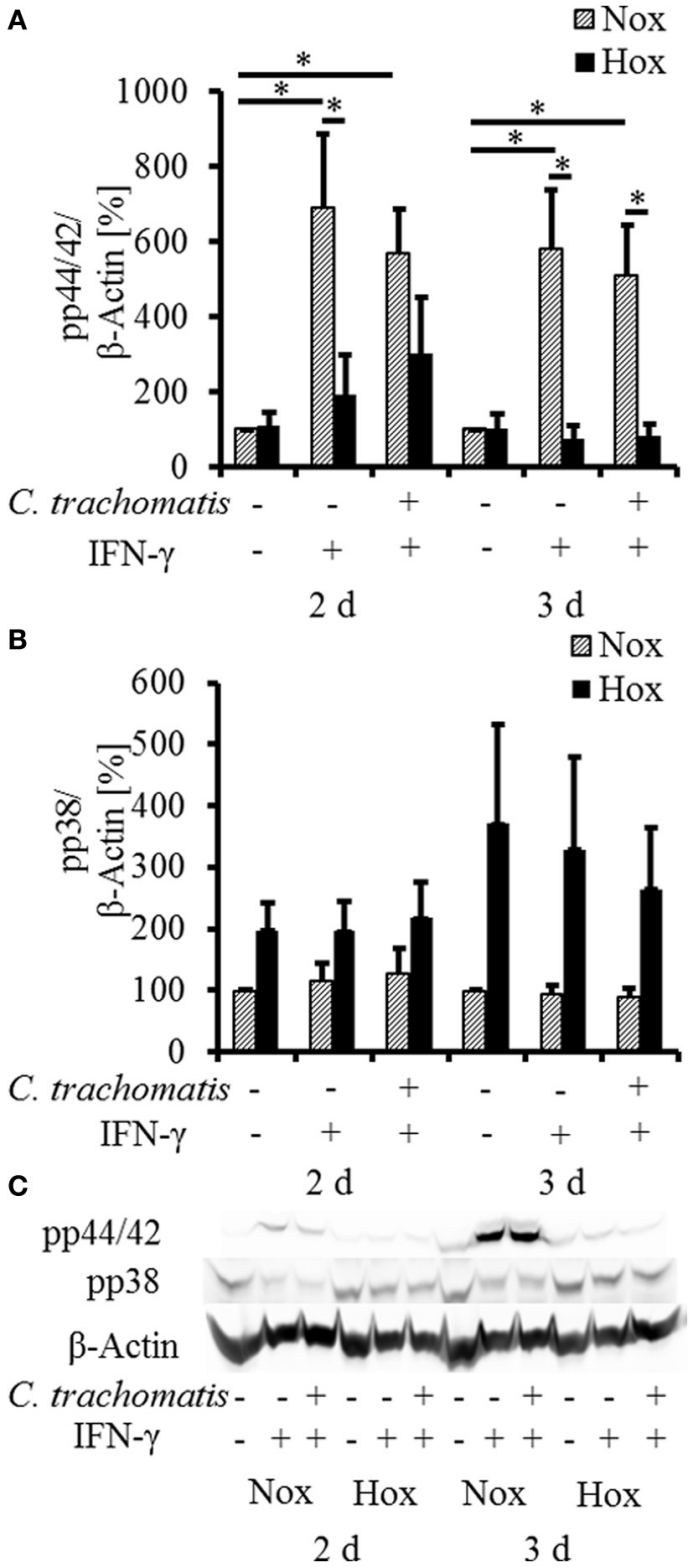
**Western blot analysis of MAP-kinase phosphorylation in IFN-γ treated *C. trachomatis* infected cells under normoxic and hypoxic conditions.** Western blot and densitometric analysis of the phosphorylation of the MAP-kinases p44/42 **(A)** and p38 **(B)** in IFN-γ treated cells after 2 and 3 d cultivation under normoxic (Nox) and hypoxic (Hox) conditions (*n* = 7, mean ± s.e.m., ^*^p ≤ 0.05). **(C)** displays a representative western blot of p38 and p44/42 phosphorylation under normoxic and hypoxic conditions.

To further investigate the influence of reactivated *C. trachomatis* on host cell immune responses under hypoxia, we analyzed the activation of the MAP-kinases p44/42 and p38 (Figure 3) which were described to be activated during productive infection under normoxic conditions. Under normoxic condition, we observed a significantly enhanced accumulation of the phosphorylated p44/42 MAP-kinase upon IFN-γ treatment which was not further enhanced in persistently infected cells. Interestingly, the accumulation of the phosphorylated p44/42 MAP-kinase was significantly reduced in IFN-γ treated cells with (Figure 3, 3 d) or without (w/o) (Figure 3, 2 and 3 d) *C. trachomatis* infection under hypoxic compared to normoxic conditions. Although hypoxic cultivation slightly enhanced the phosphorylation of p38 in all samples, no significant differences in the activation pattern were observed in IFN-γ treated cells w/o *C. trachomatis* infection. Furthermore, no accumulation of phosphorylated p38 could be observed in normoxic cells treated with IFN-γ w/o *C. trachomatis* infection.

### Reduced pro-inflammatory cytokine induction in reactivated *C. trachomatis* infection under hypoxia

**Figure 4:**
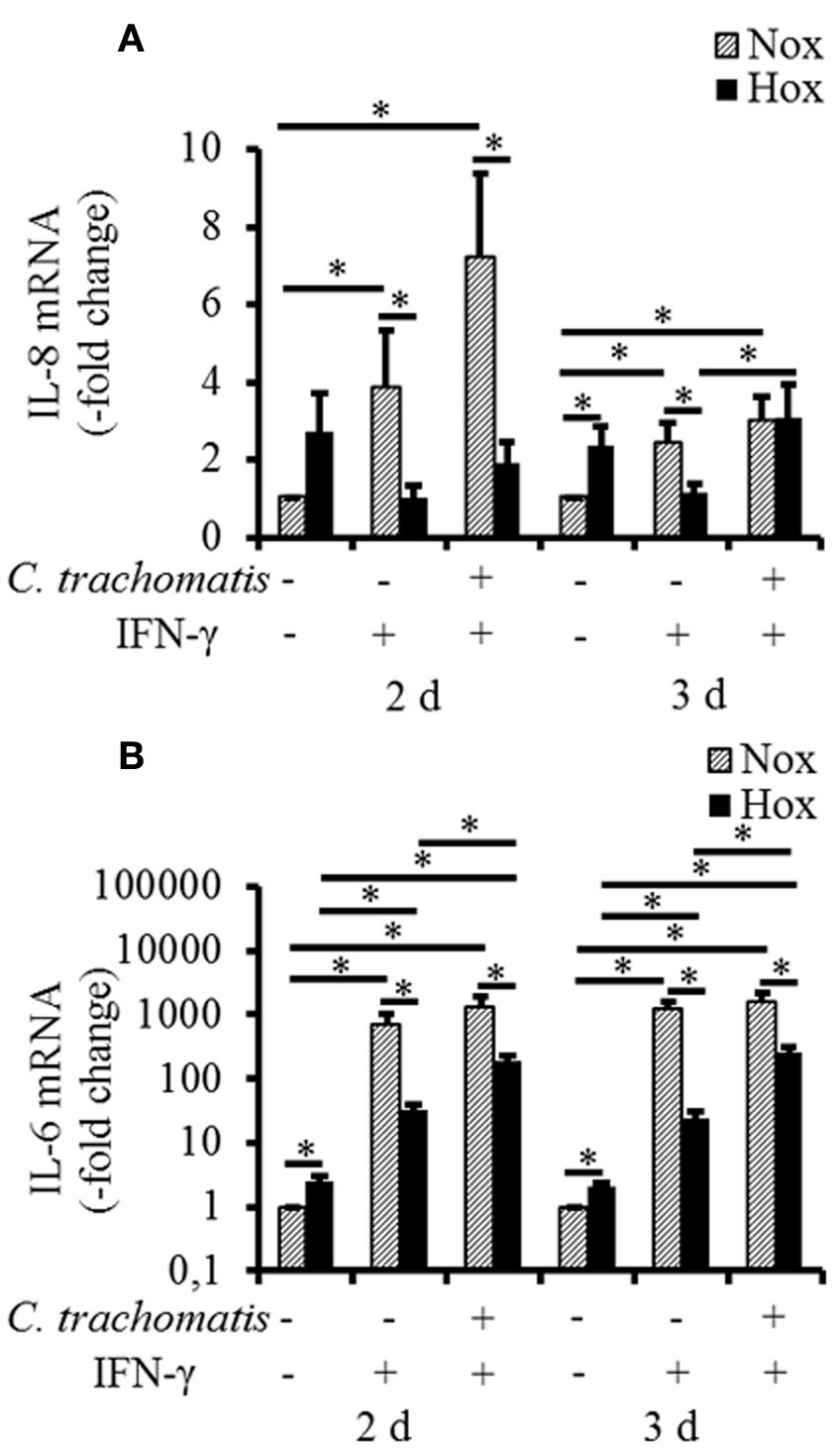
**mRNA expression of IL-6 and IL-8 in IFN-γ treated *C. trachomatis* infected cells under normoxic and hypoxic conditions.** Quantitative analysis of IL-8 **(A)** and IL-6 **(B)** mRNA expression in IFN-γ treated *C. trachomatis* infected cells after 2 and 3 d cultivation under normoxic (Nox) and hypoxic (Hox) conditions (*n* = 7, mean ± s.e.m., ^*^p ≤ 0.05).

To reveal whether hypoxic reactivation of persistent *C. trachomatis* is recognized by the host cell and translated into a pro-inflammatory cytokine response, mRNA expression levels of IL-1β, IL-6, IL-8, and MCP-1 were analyzed. Under normoxic conditions, IFN-γ treatment w/o *C. trachomatis* infection significantly up-regulated IL-6 and IL-8 mRNA expression after 2 and 3 d, respectively (Figures 4A,B). In contrast, IFN-γ alone did not (IL-8) or only moderately (IL-6) induce cytokine mRNA expression under hypoxic conditions. Besides, *C. trachomatis* infection of IFN-γ treated cells significantly up-regulated IL-6 (2 and 3 d) and IL-8 (3 d) expression compared to IFN-γ treated samples under hypoxic conditions. In all cases except for IL-8 in IFN-γ treated *C. trachomatis* infected cells after 3 d and the uninfected and untreated controls, the mRNA expression levels were significantly lower in cells that were incubated under hypoxia compared to normoxia (Figures 4A,B). For IL-1β and MCP-1 no induction of the mRNA expression levels was observed in persistently *C. trachomatis* infected cells under normoxia, nor reactivated *C. trachomatis* infection under hypoxia (Figures S1A,B). Our data indicate that in HeLa-229 cells reactivation of formerly persistent *C. trachomatis* in a hypoxic environment is accompanied by a dramatically less pronounced pro-inflammatory host cell immune response compared to persistent *Chlamydiae* under normoxic conditions.

## Discussion

Genital tract infections with the intracellular bacteria *C. trachomatis* are a frequent cause of PID, ectopic pregnancy, and tubal factor infertility (Peipert, 2003). Based on experimental data it is assumed that the infection leads via TLR or NOD1 signaling to an activation of host MAP-kinases (e.g., p44/42 and p38) as well as the NFκB pathway and subsequently to an induction of a pro-inflammatory immune response (Rasmussen et al., 1997; Hanada et al., 2003; Strober et al., 2006; Bastidas et al., 2013; Zhou et al., 2013). A mainly Th1 and NK-cell mediated release of IFN-γ protects against genital tract *C. trachomatis* infections in humans and mice (Cohen et al., 2000; Roan and Starnbach, 2006) but may also induce *C. trachomatis* persistence *in vivo* (Beatty et al., 1993). Impaired host immunity and micro- environmental conditions such as hypoxia have been shown to impair anti-chlamydial activity of IFN-γ leading to reactivation of *Chlamydiae* and finally productive infection (Roth et al., 2010).

However, nothing is known about the immune response in infected cells during reactivated *C. trachomatis* infection under hypoxic conditions. This is the first report showing that the activation of the MAP-kinase p44/42 and the expression of the pro-inflammatory cytokines IL-6 and IL-8 were diminished in reactivated *C. trachomatis* infection under hypoxia compared to persistently infected cells under normoxia. The underlying mechanisms are completely unknown but could either be attributed to oxygen-dependent host-cell signaling pathways or linked to pathogen related factors. Under normoxic conditions IFN-γ was described to induce IL-6 via activation of p44/42 (Salmenpera et al., 2003). Besides, IFN-γ prolongs the activation of p44/42 (Valledor et al., 2008) which augments the induction of IL-6 and IL-8 expression in HeLa-229 cells under normoxic conditions (Yang et al., 2008). Although a strong IFN-γ mediated activation of p44/42 and an induction of IL-6 and IL-8 was detected under normoxic conditions, less phosphorylation or cytokine induction was observed under hypoxic conditions. In accordance with previous results we observed a diminished activity of IFN-γ under hypoxia (Roth et al., 2010), which can be explained by the altered p44/42 activation under hypoxic conditions finally leading to a significantly impaired IFN-γ driven induction of IL-6 and IL-8.

Furthermore, for productively *C. trachomatis* infected cells it is known that the pathogen directly attaches to the surface of the host cell thereby activating TLR2/4 (Darville et al., 2003; Bulut et al., 2009). In addition, *C. trachomatis* may induce IL-1β, IL-6, and IL-8 by inflammasome-dependent activation of caspase-1 or NOD1 recognition (Welter-Stahl et al., 2006; Buchholz and Stephens, 2008; Cheng et al., 2008). TLR- and NOD1-mediated signaling has been directly connected to the phosphorylation of MAP-kinase p44/42 (Buchholz and Stephens, 2008; Wortzel and Seger, 2011). Under hypoxic reactivation of persistent *Chlamydiae* no activation of the p44/42 MAP-kinase was observed, which could explain the diminished IL-6 and IL-8 induction under these conditions. It was previously reported that p44/42 could be inactivated by the mitogen-activated protein kinase phosphatase-1 (MKP-1), a dual specific phosphatase carrying two hypoxic response elements (HRE) in the promoter region (Liu et al., 2005). Therefore, it has to be further elucidated if hypoxia in general dampens the p44/42 phosphorylation by MKP-1 activation and thereby modulates the immune response and cell homeostasis in a so far unknown manner. There is a tight interconnection between the NFκB and HIF-1α (hypoxia-inducible factor 1-α) signaling pathways in the regulation of inflammatory host responses (Barnes, 1997; Taylor, 2008). Thus, NFκB is activated under hypoxia (Cummins et al., 2006) but in turn also induces transcriptional up-regulation of HIF-1α expression (Rius et al., 2008). NFκB activation and subsequent pro-inflammatory gene expression is supposed to be abrogated by an enhanced expression of IκB kinase-α (IKKα) under prolonged hypoxia (Lawrence et al., 2005; Cummins et al., 2006).

Besides the above mentioned host- related factors that may dampen immune responses under hypoxia, additional mechanisms are conceivable, which are directly induced by *C. trachomatis*. Possible mediators are the chlamydial protease CT441 which might interfere with the NFκB pathway, thereby modulating the immune response and inhibiting the IL-6 and IL-8 gene expression (Lad et al., 2007a,b). For other intracellular bacteria including *Chlamydia pneumoniae* (*C. pneumoniae*), *Mycobacterium tuberculosis* (*M. tuberculosis*), and *Ehrlichia chaffeensis* (*E. chaffeensis*) several other mechanisms for silencing host immune responses under normoxia have been described. Thus, it has been shown that these bacteria are able to regulate the host immune response by impairing the TLR signaling cascade, blocking the secretion of pro-inflammatory cytokines or induction of anti-inflammatory cytokines such as IL-10 (Ismail et al., 2002; Flynn and Chan, 2003). *M. tuberculosis* expresses a 19 kDa protein with immunmodulatory functions that directly binds to the TLR2 receptor and inhibits the IFN-γ induced MHC class II antigen presentation, whereas *E. chaffeensis* was described to interfere with cytokine mRNA stability by an unknown mechanism (Lee and Rikihisa, 1996; Flynn and Chan, 2003). Degradation of the TRAF3 signaling molecule, a downstream target of TLR3, which activates IFN-β secretion, was described in *C. pneumoniae* but not in *C. trachomatis* infection (Wolf and Fields, 2013). Nevertheless, *C. trachomatis* might have similar mechanisms that upon enhanced intracellular replication after hypoxic reactivation results in the down-regulation of host immune responses.

In conclusion, IFN-γ induced persistent *C. trachomatis* is reactivated under hypoxic condition and remains mostly unrecognized by the host cell. In further experiments the influence of the impaired activation of the host immune system under hypoxia has to be elucidated in the context of the cellular metabolism and apoptosis signaling. Furthermore, the influence of hypoxia on IFN-γ induced persistence has to be clarified *in vivo*.

## Author contributions

Conception, design of the work: Stefan Jerchel, Jan Rupp; Acquisition of data: Stefan Jerchel, Larissa Schuchardt; Analysis and interpretation: Stefan Jerchel, Jan Rupp, Kensuke Shima, Inga Kaufhold, Larissa Schuchardt; Drafting the manuscript for important intellectual content: Stefan Jerchel, Jan Rupp, Kensuke Shima, Inga Kaufhold; Final approval of the version to be published: Stefan Jerchel, Jan Rupp, Kensuke Shima, Inga Kaufhold, Larissa Schuchardt; Agreement to be accountable for all aspects of the work: Stefan Jerchel, Jan Rupp, Inga Kaufhold, Kensuke Shima, Larissa Schuchardt.

## Conflict of interest statement

The authors declare that the research was conducted in the absence of any commercial or financial relationships that could be construed as a potential conflict of interest.

## Supplementary material

Figure S1 **mRNA expression of MCP-1 and IL-β in IFN-γ treated *C. trachomatis* infected cells under normoxic and hypoxic conditions.** Quantitative analysis of MCP-1 **(A)** and IL-1β **(B)** mRNA expression in IFN-γ treated *C. trachomatis* infected cells after 2 and 3 d cultivation under normoxic (Nox) and hypoxic (Hox) conditions (*n* = 7, mean ± s.e.m., ^*^*p* ≤ 0.05).
