# Supplementary figures and images for: Host immune responses after hypoxic reactivation of IFN-γ induced persistent Chlamydia trachomatis infection
Source: Front Cell Infect Microbiol. 2014 Apr 16;4:43. doi: 10.3389/fcimb.2014.00043 (PMC3997002; doi:10.3389/fcimb.2014.00043)

## Slide 1
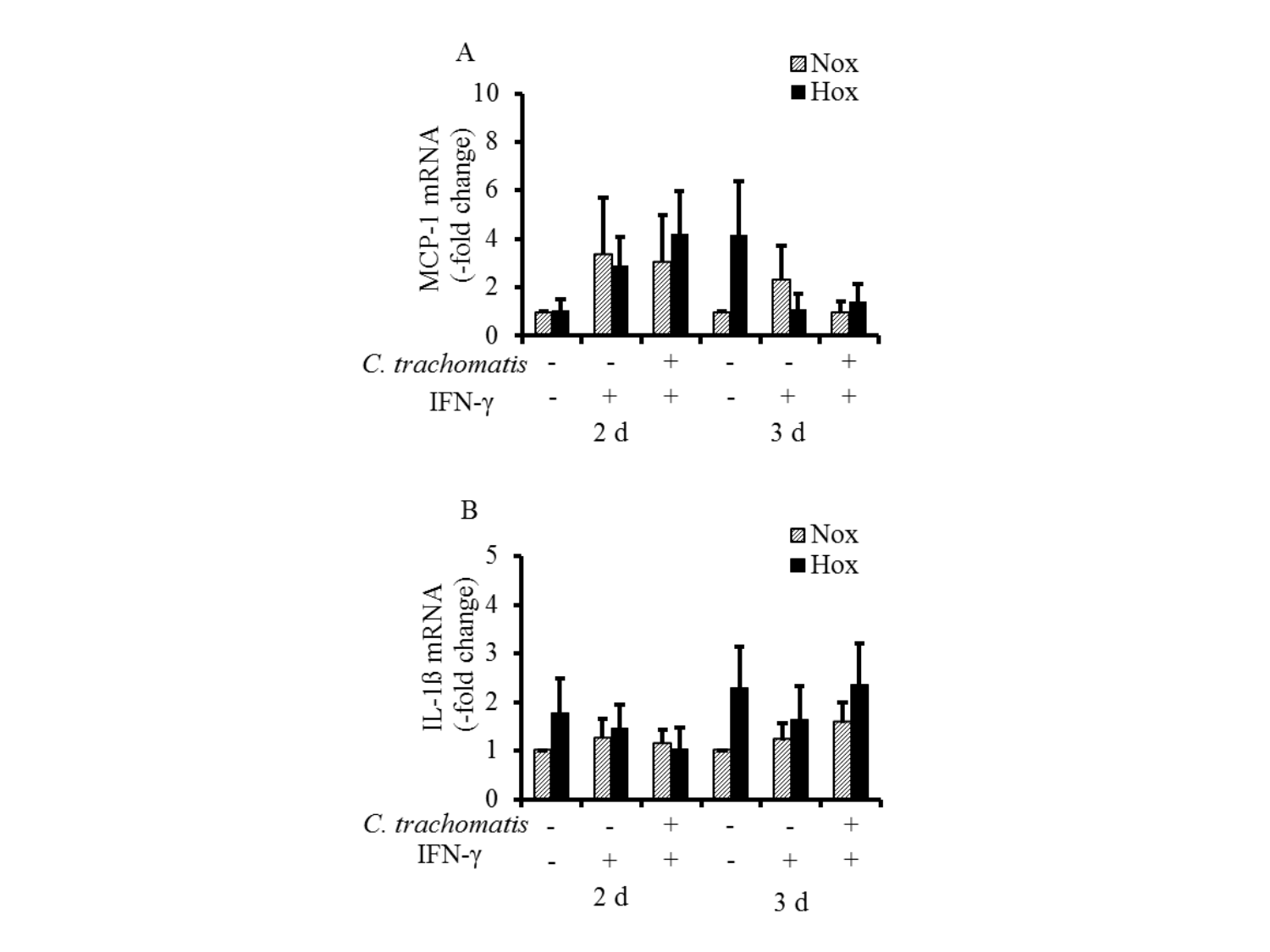

Supplement: Figure S1 — mRNA expression of MCP-1 and IL-β in IFN-γ treated C. trachomatis infected cells under normoxic and hypoxic conditions. Quantitative analysis of MCP-1 (A) and IL-1β (B) mRNA expression in IFN-γ treated C. trachomatis infected cells after 2 and 3 d cultivation under normoxic (Nox) and hypoxic (Hox) conditions (n = 7, mean ± s.e.m., *p ≤ 0.05). [file Presentation1.PPTX]
